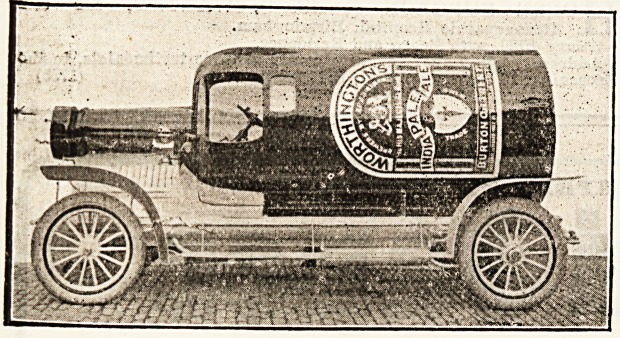# Worthington's Ales

**Published:** 1906-11-10

**Authors:** 


### WORTHINGTON'S ALES.

At the luncheon recently given at the Hôtel Cecil by Messrs. Worthington's and Co., Limited, of Burton, the firm which first established the beer industry in Burton in 1744, some interesting facts were brought out. The firm of Worthington's has grown into a vast business, and under Mr. W. P. Manners' direction it has become probably as prosperous, if not more prosperous, than any similar enterprise. Worthington's ales are deservedly famous throughout the world, and all who can appreciate a good glass of British beer owe much to the oldest firm of Burton brewers. One of the most interesting exhibits at the Brewers' Exhibition was a unique motor-car, the body of which is built in the shape of the bottle used by Messrs. Worthington and Co., Limited. We feel that this motor-car cannot fail to be of general interest, and so have pleasure in giving an illustration of it.

## Figures and Tables

**Figure f1:**